# Hydrocarbon metabolism and petroleum seepage as ecological and evolutionary drivers for *Cycloclasticus*

**DOI:** 10.1093/ismejo/wrae247

**Published:** 2024-12-18

**Authors:** Eleanor C Arrington, Jonathan Tarn, Veronika Kivenson, Brook L Nunn, Rachel M Liu, Blair G Paul, David L Valentine

**Affiliations:** Marine Science Institute, University of California, Santa Barbara, Santa Barbara, CA, 93106-9630, United States; Interdepartmental Graduate Program in Marine Science, University of California, Santa Barbara, Santa Barbara, CA, 93106-9630, United States; Innovative Genomics Institute, University of California, Berkeley, Berkeley, CA, United States; Department of Genome Sciences, University of Washington, Seattle, WA, 98195, United States; School of Oceanography, University of Washington, Seattle, WA, 98195, United States; Bay Paul Center, Marine Biological Laboratory, Woods Hole, MA, 02543, United States; Marine Science Institute, University of California, Santa Barbara, Santa Barbara, CA, 93106-9630, United States; Department of Earth Science, University of California – Santa Barbara, Santa Barbara, CA, 93106-9630, United States

**Keywords:** petroleum, hydrocarbons, metagenomics, oil spills, alkanes, comparative genomics, microbial ecology

## Abstract

Aqueous-soluble hydrocarbons dissolve into the ocean’s interior and structure deep-sea microbial populations influenced by natural oil seeps and spills. Among these hydrocarbons, *n-*pentane, is a seawater-soluble, volatile compound abundant in petroleum products and reservoirs, which partially partitions to the deep-water column following release from the seafloor. In this study, we explore the ecology and niche partitioning of two free-living *Cycloclasticus* strains recovered from seawater incubations with *n*-pentane and distinguish them as an open ocean variant and a seep-proximal variant, each with distinct capabilities for hydrocarbon catabolism. Comparative metagenomic analysis indicates the variant more frequently observed further from natural seeps encodes more general pathways for hydrocarbon consumption, including short-chain alkanes, aromatics, and long-chain alkanes, and also possesses redox versatility in the form of respiratory nitrate reduction and thiosulfate oxidation; in contrast, the seep variant specializes in short-chain alkanes and relies strictly on oxygen as the terminal electron acceptor. Both variants observed in our work were dominant ecotypes of *Cycloclasticus* observed during the Deepwater Horizon disaster, a conclusion supported by 16S rRNA gene analysis and read-recruitment of sequences collected from the submerged oil plume during active flow. A comparative genomic analysis of *Cycloclasticus* across various ecosystems suggests distinct strategies for hydrocarbon transformations among each clade. Our findings suggest *Cycloclasticus* is a versatile and opportunistic consumer of hydrocarbons and may have a greater role in the cycling of sulfur and nitrogen, thus contributing broad ecological impact to various ecosystems globally.

## Introduction

Much of the petroleum entering the ocean annually is introduced near the seafloor from human-caused incidents such as pipeline ruptures, well blowouts, and leaking submerged oil tankers, alongside other deep hydrocarbon inputs originating from natural oil seepage and hydrothermal vents. Following petroleum release to the seafloor, several compounds dissolve into seawater due to their aqueous solubility, subsequently affecting the microbial community within the surrounding water column [1–3]. These semi-aqueous soluble compounds can be overlooked as drivers of microbial metabolism in the deep community because these compounds often evaporate from surface oil slicks exposed to the atmosphere, which receive the majority of attention from agencies and scientists responding to oil-related incidents. This work focuses on the semi-aqueous-soluble compound *n-*pentane, which is known to partition to the deep ocean following release from the seafloor [2, 3].

Petroleum exposure to seawater substantially decreases prokaryotic diversity due to a strong selection for hydrocarbon-degrading microorganisms and toxic effects on other taxa [4, 5]. Models of in-situ hydrocarbon biodegradation indicate that as a water parcel encounters a hydrocarbon source, a seed population of hydrocarbon degraders grows abundantly [6–11]. The origin and ecology of these seed populations are primarily hypothetical. Many studies have suggested seed populations are prolonged and sustained by hydrocarbon substrates originating from various sources, including cyanobacteria and eukaryotic phytoplankton [12–14], as well as natural gas seepage and hydrothermal vents [15, 16]. As an example, the ubiquitous alkane degrader, *Alcanivorax*, exhibits basal cell populations that range from 10 to 5000 cells per ml in uncontaminated seawater [6, 17], with recent work suggesting high native abundance is subsidized by widespread biosynthesis of long-chain *n*-alkanes by marine phytoplankton [13, 18]. Other recent evidence has shown that methanotrophs can be physically transported on bubbles from a gas seep [19, 20], pointing to seeps as a physical mechanism to seed the water column with hydrocarbon degraders. Alternatively, facultative hydrocarbon degraders could be present that rely on other metabolic inputs such as amino acids, carbohydrates, lipids, or other organic acids and switch to hydrocarbons under appropriate conditions [21–23]. Very few studies have focused on how these factors control the development of a petroleum-degrading community during oil spills in previously uncontaminated waters.

**Figure 1:**
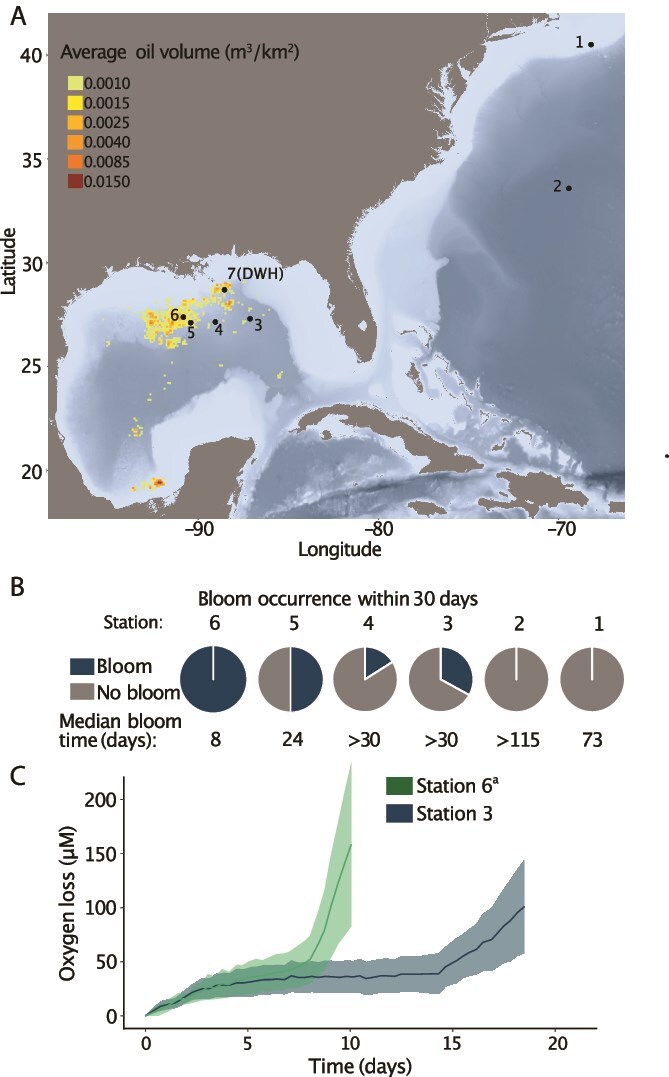
Previously published data in [18]. *n*-pentane biodegradation informed by respiration after normalization to unamended controls. (**A**) stations relative to natural petroleum seepage. Origin of natural seep locations from [24]. DWH indicates the location of the sample collected during the Deepwater Horizon (DWH) disaster and sequenced in this study. (**B**) fraction of incubations that met the operational definition of a bloom (three consecutive time points were observed with oxygen loss greater than 0.21 μM) and median time bloom occurred (*n* = 6 at stations 3–6, n = 8 at stations 1 and 2) [18]. Median bloom times over the entire experiment, assuming all bottles bloom given enough time.  Duration of experiement varied from 27 d to 30 d in the GOM and 115 d in the North Atlantic. (**C**) oxygen loss in *n-*pentane incubations with time for blooms at Station 6 and Station 3. (a) indicates that one incubation bloom (sample 180) was omitted as it was an outlier with delayed bloom response (23 days) compared to the other five replicates which bloomed in 8 days.

Our investigation [18] of the ocean’s biological hydrocarbon cycle revealed the microbial response to *n-*pentane is structured by proximity to seepage (Fig. 1). This previous work and our current study focus on sea-going incubations conducted with water collected from the deep ocean (1000 m) along a transect spanning the Gulf of Mexico (GOM) and the North Atlantic. *n-*Pentane metabolism was observed through a closed-system optical oxygen technique. Blooms were designated as present when three consecutive time points exhibited oxygen loss greater than 0.21 μM h^−1^ after normalization to unamended controls. This definition was based on the observation that each incubation meeting this threshold continued to exhibit oxygen decline at this rate or higher until near-hypoxic conditions were reached, indicating a bloom-like state. We observed distinct bloom response times to *n*-pentane in relation to natural seepage, whereby bloom onset on *n-*pentane is ~9X faster in the seep-ridden Northwest GOM compared to the water underlying the North Atlantic subtropical gyre [18, 24]. Median bloom times varied from 72.9 days furthest from natural seepage to 8.3 days closest to natural seepage. The fraction of samples that exhibited a bloom response also coincided with proximity to natural seepage, with 100% of incubations blooming within 30 days near seepage, 33% blooming further from seepage in the northeastern GOM, and 0% of incubations blooming outside the Gulf within that same timeframe (Fig. 1B). This previous work focused on the timing and occurrence of respiratory blooms on *n-*pentane but did not explore which microorganisms were responsible for *n*-pentane consumption or how natural seepage influences which microorganism responds in each of these settings.

Here, we investigate the influence of biogeography on microbial hydrocarbon metabolism by analyzing the genomic content of organisms with contrasting bloom responses and source water origins within the GOM. We extract and analyze the 16S rRNA gene content of blooms, assemble high-quality draft metagenome assembled genomes (MAGs) from bloom experiments, and conduct complementary proteomic analysis to evaluate metabolism. The predominant microbial member of the *n-*pentane-enriched community across the GOM belongs to the *Cycloclasticus* genus, initially named for their metabolic capability to consume polycyclic aromatic hydrocarbons (PAHs). *Cycloclasticus*, a member of the class *Gammaproteobacteria,* is often detected in oil-rich oceanic regions [25–27]. *Cycloclasticus* has also been found as a symbiont in the tissues of mussels and sponges at deep-sea oil seeps, where they likely metabolize short-chain alkanes [15]. All cultures of *Cycloclasticus* have been isolated on aromatic substrates and are closely related according to 16S rRNA gene phylogeny [15, 25, 28–30]. We observe two strains of *Cycloclasticus* bloom in response to short-chain alkanes in the GOM, with one strain favoring the seep-influenced region of the northwestern GOM (hereafter referred to as the seep variant [SV]). In contrast, the other strain favors the open ocean region far from natural seepage (hereafter referred to as the open ocean variant, OOV).

## Materials and methods

### Incubation design and sample collection

Seawater samples were collected on two research cruises aboard RV Atlantis in June 2015 and the RV Neil Armstrong in May 2017. *n-*Pentane incubations were conducted at stations 1 (40° 9.14′ N, 68° 19.889′ W), 2 (33° 58.21′ N, 69° 43.38′ W), 3 (27° 30.41′ N, 87° 12.41′ W), 4 (27° 15.00′ N, 89° 05.05′ W), 5 (27° 11.60′ N, 90° 41.75′ W), and 6 (27° 38.40′ N, 90° 54.98′ W) with seawater collected from 1000 m. Respiration data and methods are available from [18], with sample numbers re-named for this study to exclude irrelevant data. Seawater collected from CTD Niskin bottles was transferred into 250 ml glass serum vials using a short length of Tygon tubing. The vials were overflowed with at least 3 volumes of water, ensuring no air bubbles were present, before sealing with polytetrafluoroethylene (PTFE) coated chlorobutyl rubber stopper and crimp cap. All bottles, except for unamended blank controls, immediately received 10 μl of *n-*pentane using a gas-tight syringe (Hamilton) and were maintained in the dark at in-situ temperature (4°C). Before filling, each serum bottle was fixed with a contactless optical oxygen sensor (Pyroscience) on the inner side with silicone glue, and afterward were cleaned from organic contaminants with triple rinses of ethanol, 3% hydrogen peroxide, 10% hydrochloric acid, and MilliQ water, and were sterilized via autoclave. Oxygen concentration was monitored approximately every 8 h with a fiber optic oxygen meter (Pyroscience). Observed changes in oxygen content were normalized to unamended controls to correct for oxygen loss from background respiration processes and variability due to temperature changes. After 27–30 days, each incubation was sacrificially harvested, samples were collected for nutrient analysis and cell count analysis, and the remaining seawater was filtered on a 0.22 μm polyethersulfone filter and stored at −80°C until further analysis.

### Nutrient and cell enumeration

Before filtration, seawater was collected from incubations for cell enumeration via flow cytometry and nutrient analysis. 2 ml subsamples for prokaryotic cell abundance were fixed with 0.2% paraformaldehyde and quantified using the Millipore Guava EasyCyte 5HT flow cytometer as in [31]. The dissolved nutrient (nitrate, phosphate, and ammonia) sample collection was conducted following the requirements of the University of California, Santa Barbara Marine Science Institute Analytical Lab. Seawater incubation samples were filtered through a 0.2 μm polyvinylidene (PVDF) filter into triple-rinsed plastic HDPE 20 ml vials. Nutrient sample volumes were ∼17 ml water and stored frozen at −20°C until analysis. Dissolved nutrient concentrations were analyzed by flow injection analysis using the QuikChem 8500 Series 2 (Lachat Instruments).

### Deepwater Horizon archival sample

We extracted and analyzed two replicate archived environmental DNA samples collected from the Deepwater Horizon event on May 30th, 2010, at a depth of 1090 m, while the wellhead was still leaking into the GOM. When samples were originally collected, microbial biomass was filtered onto 0.2-micron Sterivex filters (Millipore) and stored at −80°C until further analysis.

### DNA extraction, PCR amplification, and 16S rRNA gene analysis

DNA was analyzed from stations within the GOM and the DWH archival samples. DNA extraction was performed from ¼ of each filter using the PowerSoil DNA extraction kit with the following modifications: 200 μl of bead beating solution was removed at the initial step and replaced with phenol-chloroform, the C4 bead binding solution was supplemented with 600 μl of 100% ethanol, and we added an additional column washing step with 650 μl of 100% ethanol. Extracts were purified and concentrated by ethanol precipitation, then stored at −80°C. The V4 region of the 16S rRNA gene was amplified, and each sample was barcoded as previously described [32] with the 515F-Y and 806RB primers as previously published [33–35]. Amplicon PCRs contained 1 μl of template DNA, 2 μl of forward primer, 2 μl of reverse primer, and 17 μl of AccuPrime Pfx SuperMix. Thermocycling conditions consisted of 95°C for 2 min, 30 cycles of 95°C for 20 s, 55°C for 15 s, 72°C for 5 min, and a final elongation at 72°C for 10 min. Sample DNA concentrations were normalized using the SequelPrep Normalization Kit, cleaned using the DNA Clean and Concentrator kit, visualized on an Agilent Tapestation, and quantified using a Qubit Fluorometer. Samples were sequenced at the UC Davis Genome Center on the MiSeq platform (Illumina) with 250 nucleotide paired-end reads. A PCR-grade water sample was included in extraction, amplification, and sequencing as negative control to assess for DNA contamination.

Trimmed fastq files were quality filtered using the fastqPairedFilter command within the dada2 R package, version 1.9.3 [36] with following parameters: truncLen = c(190,190), maxN = 0, maxEE = c(2,2), truncQ = 2, rm.phix = TRUE, compress = TRUE, multithread = TRUE. Quality filtered reads were dereplicated using derepFastq command. Paired dereplicated fastq files were joined using the mergePairs function with the default parameters. A amplicon sequence variant (ASV) table was constructed with the makeSequenceTable command, and potential chimeras were removed denovo using removeBimeraDenovo. Taxonomic assignment of the sequences was done with the assignTaxonomy command using the Silva taxonomic training dataset formatted for DADA2 v132 [37, 38]. If sequences were not assigned to the Silva database, they were left as NA.

### Metagenomic sequencing and reconstruction

Metagenomic library preparation and high-throughput sequencing were conducted at the University of California Davis DNA Technologies Core. DNA was sequenced on the HiSeq4000 (Illumina) platform, producing 150-base pair (bp) paired-end reads with a targeted insert size of 400 bp. Quality control and adaptor removal were performed with Trimmomatic [39] (v.0.36; parameters: leading 10, trailing 10, sliding window of 4, quality score of 25, minimum length 151 bp).

10–70% of the trimmed high-quality reads were randomly subsampled to deconvolute assembly and downstream binning in samples with very high coverage, as in [40]. Subsamples of each metagenomic dataset were tested in increments of 10% to determine which percentage produced the highest quality *Cycloclasticus* MAG based on completion, redundancy, and number of scaffolds in the genome. The exception is sample “Cycloclasticus_sp_3_C5_1”, which was tested in increments of 5% subsampled reads to reduce the number of scaffolds in the MAG to 1. The final subsampled percentage for each sample is noted in Supplementary Dataset S4. The program dRep was used to dereplicate all MAGs created from each subsampled dataset using default parameters, which group genomes based on initial 90% MASH (MinHash distance) clustering and 95% average nucleotide identity [41]. Only one dereplicated MAG was recovered from each sample except the DWH sample.

The subsampled high-quality reads were assembled using metaSPAdes [42] (v.3.8.1; parameters *k* = 21, 33, 55, 77, 88, 127). The quality of assemblies was determined using QUAST [43] (v.5.0.2 with default parameters). Sequencing coverage was determined for each assembled scaffold by mapping high-quality reads to the assembly using Bowtie2 [44] (v.2.3.4.1; default parameters) with Samtools [45] (v.1.7). Contigs greater than 2500 bp were manually binned using Anvi’o with Centrifuge (v.1.0.1) based on coverage uniformity and GC content (v.5) [46, 47]. Quality metrics for metagenome-assembled genomes (MAGs) were determined using CheckM [48] (v.1.0.7; default parameters). The taxonomy of each MAG was classified using GTDB-Tk (v.1.0.2) against The Genome Taxonomy Database [49] (https://data.ace.uq.edu.au/public/gtdb/data/releases/release89/89.0/, v.r89). The average nucleotide identity of each genome was determined with the ANI Matrix via the Enveomics tool collection [50].

**Figure 2:**
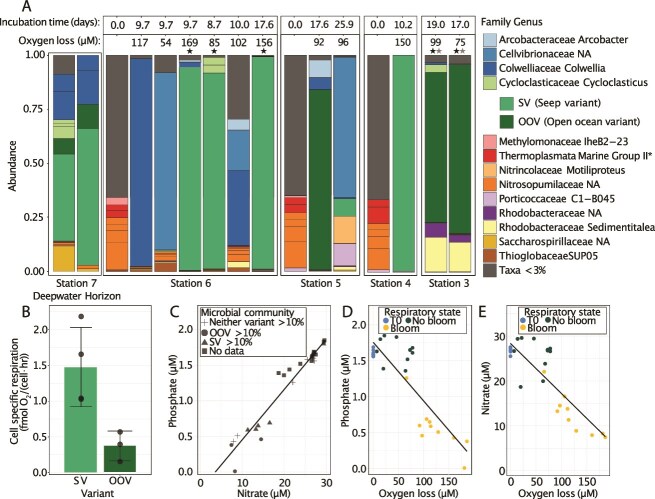
Microbial and geochemical characteristics of *n*-pentane blooms alongside environmental samples from the Deepwater Horizon event. (**A**) microbial community composition informed via the V4 region of the 16S rRNA gene. Initial “T0” samples collected at the start of incubations are denoted with an incubation time of 0.0 days. *n*-Pentane incubations were harvested after meeting the operational definition of bloom state (three consecutive time points were observed with oxygen loss greater than 0.21 μM). ‘Oxygen loss’ refers to the change in oxygen levels relative to initial concentrations, after normalizing to loss in unamended controls, at the point when the DNA sample was collected. Black and gray stars indicate metagenomic and metaproteomic samples, respectively. SV strain *Cycloclasticus* blooms in ~9–18 days in locations close to natural seepage and was abundant during the Deepwater Horizon disaster. OOV strain of *Cycloclasticus* blooms in 17–19 days and dominates seawater originating further from natural seepage inputs. ^*^No taxonomic representative at the family or genera level. (**B**) cell-specific respiration in incubations dominated (>80%) by SV and OOV *Cycloclasticus*. (**C**) dissolved phosphate vs dissolved nitrate concentration in initial samples (T0) and at sample harvest. (**D**) dissolved phosphate concentration in final samples is depleted compared to unamended controls and initial samples (T0). (**E**) dissolved nitrate concentration in final samples is depleted compared to unamended controls and initial samples (T0).

We reconstructed high-quality MAGs from five pentane bloom samples, with completeness >97% and redundancy <2% (black stars in Fig. 2). Three MAGs, named “6_C5_1”, “6_C5_2”, and “6_C5_3”, originated from Station 6 (natural seep region), and two MAGs, named “3_C5_1” and “3_C5_2”, originated from Station 3 (open ocean region). Based on the 16S rRNA gene analysis of the two DWH samples from this study, two variants of *Cycloclasticus* were present in the sample sequenced for metagenomics. For the DWH metagenome, the second variant related to OOV could not be recovered with metagenomics due to issues with assembly fragmentation and binning of the two closely related strains. To obtain a high-quality draft MAG of “MAG_DWH_1”, we subsampled our reads by 50% until the OOV-related sequences were a small fraction of the assembled data.

The program dRep was used to dereplicate all MAGs reconstructed in this study using default parameters. dRep groups genomes based on initial 90% MASH (MinHash distance) clustering and 95% average nucleotide identity [41]. This analysis created two clusters of essentially identical genomes: with “6_C5_1”, “6_C5_2”, and “6_C5_3” in one cluster and another containing “3_C5_1” and “3_C5_2”. We also tested whether automated binning with MetaBAT2 [51] would alter the resulting MAGs but found that dRep also clustered MAGs derived from the two different binning methods as essentially identical. We further note each MAG was recovered from biologically independent incubations, yet every component of metabolism and taxonomic marker analyzed was nearly identical within each ecotype variant; therefore, we will refer to “SV-MAG” as the three MAGs from Station 6 and “OOV-MAG” as the two MAGs from Station 3. We also downloaded single amplified genomes (SAGs) from the Joint Genome Institute that originated from the Deepwater Horizon event under the accession numbers 2599185270, 2599185276, and 2599185294.

### Other metagenomic reconstructions

Using a 16S rRNA gene search tool through the Joint Genome Institute - Integrated Microbial Genomes and Microbiomes (JGI-IMG) portal, we identified public environmental metagenomic datasets with *Cycloclasticus* representation. These datasets were downloaded, and metagenomic reconstruction was performed according to the above protocol with the following modifications: binning was performed using the automated binning software MetaBAT2 [51]. Each *Cycloclasticus* MAG recovered was manually refined with Anvi’o based on coverage uniformity and GC content (v.5), [46, 52]. See the acknowledgments section regarding the origin of the Groves Creek Salt Marsh MAGs.

### Annotation

Open reading frames were predicted for MAGs using Prodigal [53] (v.2.6.3; default parameters). Functional annotation was determined using HMMER3 [54] (v.3.1b2) against the Pfam database [55] (v.31.0) and TIGRFAM database [56] (v.15) with an expected value (e-value) cutoff of 1 × 10^−7^, as well as the KofamScan (v.1.1.0) [57] against the Hidden Markov model (HMM) profiles for Kyoto Encyclopedia of Genes and Genomes and Kegg Orthology (KEGG/KO) with the adaptive score thresholds associated with each KO.

### Phylogenetics

To define genome phylogenomic relationships of MAGs, 16 universal ribosomal proteins (RPs) were used L2-L6, L14-L16, L18, L22, L24, S3, S8, S10, S17, and S19. This dataset was not dereplicated using dRep to show variability in metabolism among closely related *Cycloclasticus* MAGs/genomes. For phylogenies of metabolic genes and RPs, all representative sequences and concatenated alignments containing <25% informative sites were excluded in tree construction. For phylogenetic trees of the PQQ-dependent alcohol dehydrogenase protein family, 16S rRNA gene, DMSO protein superfamily, and the copper-bound membrane monooxygenase (CuMMO) protein superfamily, all sequences used are in Supplementary Dataset S6 and Supplementary Dataset S7. Genomes under “genome accession” were downloaded from NCBI or JGI and annotated according to the above “Annotation” section. The 16S rRNA genes were detected from the *Cycloclasticus* genome/MAG collection with RNAmmer [58]. Among the collection of genomes used in this study, accession “TIGR03080” was used to find CuMMO/particulate hydrocarbon monooxygenase proteins, “TIGR01580” was used to find *narG* proteins, and the accession “PF01011” was used to find PQQ-dependent alcohol dehydrogenase proteins. In all phylogenetic trees, amino acid sequences were aligned using MUSCLE (v.3.8.425) [59]. All columns with >95% gaps were removed using trimAl [60]. Maximum-likelihood phylogenetic analysis of concatenated alignment was inferred by RAxML [61] (v.8.9; parameters: raxmlHPC -T 4 -s input -N autoMRE -n result -f a -p 12345 -x 12 345 -m PROTCATLG). The resulting trees were visualized using FigTree [62] (v.1.4.3).

### Metaproteomics

We analyzed metaproteomes from two of the OOV (open ocean variant) samples with corresponding MAGs (“3_C5_1” and “3_C5_2”) as the reference databases. Proteins from each sample were extracted and prepared from ¼ filter (equivalent to ~60 ml of filtered water and ~1.3x10^8^ bacteria) for liquid chromatography and tandem mass spectrometry (LC–MS/MS) using a protocol adapted from [63]. Briefly, filters were cut into 2 mm pieces and submerged in 100 μl of 6 M urea and 600 μl of 50 mM NH_4_HCO_3_ and sonicated with a Branson 250 Sonifier; 20 kHz, 5 × 20 s on ice to lyse cells. Protein concentrations for each sample were quantified in triplicate using a Bicinchoninic Acid protein assay kit (Pierce Thermo Scientific) using a microplate reader. Proteins within the lysate were reduced and alkylated using dithiothreitol and iodoacetamide, respectively, digested with Trypsin (12 h; 1:20 enzyme to protein) and desalted with C18 centrifugal spin columns. Peptides were dried down and resuspended in 2% ACN, 0.1% formic acid before analysis with a nanoAcquity UPLC (Waters Corp., Milford, MA) in line with a Q-Exactive-HF (Thermo Fisher Scientific, Waltham, MA). Reverse phase chromatography was achieved using a PicoTip (New Objective) fused silica capillary column (75 μm i.d., 30 cm long) packed with C18 beads (Dr. Maisch ReproSil-Pur; C18-Aq, 120 Å, 3 μm). The analytical column was preceded by a 150 μm i.d. PicoFrit (New Objective) precolumn packed with C-18 beads to 3 cm long (Dr. Maisch ReproSil-Pur; C18-Aq, 120 Å, 3 μm). Peptides were eluted using a 90-min acidified (formic acid, 0.1% v/v) water-acetonitrile gradient (2%–45% acetonitrile).

Sample analyses on the MS were randomized to reduce batch effects, and quality control (QC) peptide mixtures were analyzed every six injections to monitor chromatography and MS sensitivity. Each sample was analyzed with data-dependent acquisition (DDA). From precursor ion scans of 400–1200 m/z the top 20 most intense ions were selected for MS2 acquisition. Centroid full MS resolution data was collected at 70000 with AGC target of 1 × 106 and centroid MS2 data was collected at resolution of 35 000 with AGC target of 5 × 104. Dynamic exclusion was set to 20 s and + 2, +3, +4 ions were selected for MS2 using DDA mode. Comet [64, 65] was used to search the DDA files against the two MAGs concatenated with 50 common contaminants and the QC peptides. Comet parameters included: 10 ppm precursor mass tolerance, fully-tryptic specificity with 0 allowed missed cleavages, cysteine modification of 57 Da and modifications on methionine of 15.999 Da. PeptideProphet was used to validate peptide spectral matches and determine thresholds for a false discovery rate [66]. All relevant peptide hits with an e-value less than 0.01 were used to define protein presence. All peptides’ tandem mass spectra discussed here were manually investigated to verify b and y ions.

### Read-recruitment analysis

To better understand the biogeography of OOV-MAG and SV-MAG, we searched a representative genome from each variant against the Branchwater web interface to learn which datasets among millions of global metagenomes indexed by Branchwater contain matches filtered to 0.97 cANI to either genome [67]. All projects matching OOV-MAG or SV-MAG were downloaded and trimmed (according to the above parameters). To evaluate the prevalence of *Cycloclasticus* MAGs and SAGs across these metagenomic datasets (including the Deepwater Horizon event), we first dereplicated the genomes/MAGs with stringency of 95% average nucleotide identity using dRep [41], then used Bowtie2 for read mapping [44] (v.2.3.4.1; default parameters) and analyzed the mapped reads using stringent parameters from InStrain [68]; namely we filtered reads based on 92% similarity and only noted a genomes’ presence when they achieved >20% breadth (meaning at least 20% of the genome was detected), and the expected breadth was within 20% of the observed breadth (indicating the reads were mapped randomly across the genome).

## Results and discussion

### Variant biogeography

Incubations conducted with seawater from 1000 m depth containing ambient nutrients successfully exhibited robust blooms of bacteria when supplied with *n-*pentane as a carbon and energy source. Blooms are characterized by bacterioplankton abundance, increasing by ~10X compared to unamended controls (Supplementary Dataset S1), drawdowns in inorganic phosphate and nitrate concentration (Fig. 2C, Fig. 2D, Fig. 2E; Supplementary Dataset S1), and the emergence of dominant taxa comprising >70% of the microbial community at the termination of the incubation (Fig. 2A). Community analysis of incubations that failed to bloom within the experimental timeframe (27–30 days) revealed instances where the community was dominated by a limited number of taxa, indicating a microbial community shift precedes major respiratory signals (Supplementary Dataset S2, Supplementary Information Fig. S3). Over 60% of all *n-*pentane blooms in the deep GOM were dominated by the *Cycloclasticus* genus (Fig. 2A). Variation among replicate incubations was observed as a change in the dominant taxa, which occurred more often at stations closer to natural seepage, potentially related to greater microbial diversity and abundance of alkane degraders in those waters. At Station 6, *Colwellia* and an unclassified genus belonging to *Cellvibrionaceae* bloomed, and the same unclassified *Cellvibrionaceae* bloomed at Station 5 (Fig. 2A).

Our data show a difference in the frequency of incubations that bloom between Station 3, located further from seeps, and Station 6, situated within a dense seep field. At Station 3, only two out of six replicates bloomed within 30 days, whereas at Station 6, all six replicates bloomed within the same timeframe. Our interpretation of these data is *n-*pentane degraders are less concentrated in seawater collected further from seeps, leading to inconsistent bloom patterns. Even though Station 5 is among the seep fields in the Northwestern GOM (Fig. 1A), it also has an inconsistent bloom response, with only 50% of incubations blooming within 30 days. This may be explained by the western flow of eastern sourced seep-deplete waters observed at the time seawater was collected (Supplementary Data Fig. S7). Collectively, bloom kinetic data and the direction of deep ocean currents indicate Station 6 likely experienced a strong and recent influence from natural seepage.

Among the blooms, we identified two dominant *Cycloclasticus* variants. One, called the SV, was the primary blooming population at Station 6, located within the northwestern GOM seep field. The other, termed the “open-ocean variant” (OOV), was the primary blooming organism at Station 3, the more offshore petroleum-depleted region (Fig. 1A, Fig. 2A, Supplementary Data S3). The distribution of SV and OOV was more varied at Stations 4 and 5, with both variants present at Station 4, but only OOV blooming at Station 5 (Fig. 2A). In incubations enriched with *n*-pentane and sequenced for 16S rRNA gene analysis—regardless of bloom status—OOV was more than 10% abundant in five of eight incubations from Stations 3–5 (Supplementary Information, Fig. S3), indicating its numerical dominance in areas with patchy bloom patterns. In contrast, SV was detected in only two of the eight incubations. Although referred to as 'SV, seep variant' and 'OOV, open-ocean variant,' both variants may occur across these environments, with numerical dominance observed in their respective settings. A read-recruitment analysis of SV-MAG and OOV-MAG distribution across the GOM further supports this interpretation (Supplementary Dataset S5 and Supplementary Information Fig. S9).

Cell-specific respiration is higher for SV than the OOV (Fig. 2B). The respiration profile (Fig. 1C) of these variants also showed distinct patterns, where OOV presents more gradual oxygen consumption with time. The OOV was also observed in small relative abundances (<1%) at Station 6 (Supplementary Dataset S2). In each *n-*pentane incubation where the two variants co-occurred, the SV numerically dominated by ~3 orders of magnitude except for the *Colwellia*/*Cellvibrionaceae* bloom at Station 6, where both variants were detected at <1% abundance. This suggests that SV is better adapted to conditions associated with natural seepage, as it outcompeted OOV, which only bloomed at stations that are distant from seeps.

### Pentane metabolism

**Figure 3:**
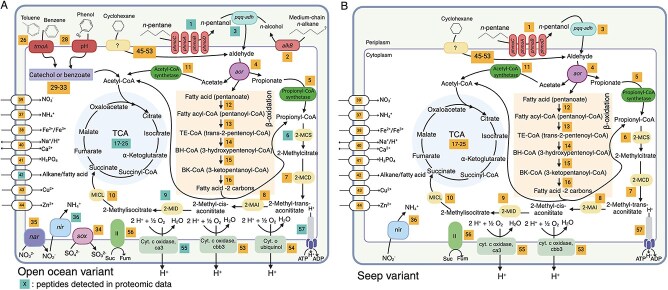
Carbon, nitrogen, and sulfur metabolism present in *Cycloclasticus* variants blooming on *n-*pentane. (**A**) open ocean variant, OOV-MAG, and (**B**) seep variant SV-MAG. Yellow boxes indicate a reaction (and its reference number) that could be linked with a predicted metabolic function, see Supplementary Dataset S3. Blue boxes indicate peptides for that enzyme were observed in proteomic data. Proteomics performed soley on OOV-MAG. If the reaction box describes multiple enzymes, only one needs to be observed in proteomic data for it to be colored blue. Enzyme abbreviations: particulate hydrocarbon monooxygenase *phmo* (A, B, C, D); PQQ-dependent alcohol dehydrogenase (*pqq-adh*); aldehyde oxidoreductase (aor); 2-methylcitrate synthase (2-*mcs*); 2-methylcitrate dehydratase (2-MCD); 2-methylcitrate isomerase (2-MAI); 2-methylisocitrate dehydratase (2-MID); methylisocitrate lyase (MICL); nitrite reductase (*nir*); respiratory nitrate reductase (*nar*); thiosulfate oxidation complex (*sox*); alkane-1-monooxygenase (*alkB*); toluene monooxygenase (*tmoA*); phenol/toluene 2- monooxygenase (pH). The tricarboxylic acid (TCA) and beta-oxidation pathway are highlighted in blue and peach colors.

**Figure 4:**
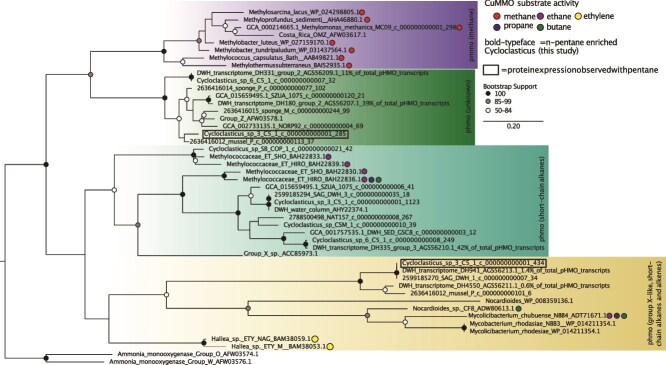
Maximum likelihood tree of *phmo* subunit a drawn to scale, with branch lengths representing the number of substitutions per site. Bootstrap values below 50% are not shown. Each major clade is labeled according to substrate affinity of experimentally validated sequences within that clade. From top to bottom: the purple clade represents *pmmo* with activity on methane, the next clade (green) lacks any known substrate specificity and is thus labeled “unknown”, the cyan clade represents group X *phmo* genes (ethane/ethylene, propane, and butane activity), and the bottom yellow clade are group-X like (ethane, propane, and butane activity). Sequences from the *n*-pentane-enriched *Cycloclasticus* MAGs are in bold; boxed values indicate *phmo* gene detected in proteomic data.

Within both SV- and OOV-MAGs, we found genomic potential for *n-*pentane utilization for catabolism and anabolism (Fig. 3). Our analyses were further supported by proteomic analysis of the OOV-MAG from Station 3 (analysis performed on samples with gray stars in Fig. 2A; results in Fig. 4B). The first step in the consumption of *n-*pentane is the oxidation to pentanol, and we hypothesize that this step is catalyzed by the copper-containing membrane-associated monooxygenase, called particulate hydrocarbon monooxygenases (*phmo*). The most well-characterized *phmo* is the particulate methane monooxygenase, which oxidizes methane to methanol [20]. *phmo* has never been demonstrated to act on *n-*pentane, though it has shown activity on *n-*butane in previous work [69]. We found multiple copies of genes encoding *phmo* in both *Cycloclasticus* MAG variants (Fig. 3, Fig. 4, Supplementary Information Fig. S8).

Each copy of *phmo* varies phylogenetically from the other copies within the same MAG, suggesting each operon may have different substrate specificities or capitalize on alkanes of varying substrate concentrations [70] (Fig. 4). Both MAG-SV and MAG-OOV have *phmo* sequences that form monophyletic clades with reference sequences with demonstrated affinity for ethane and butane. Both variants also contain a sequence that forms a monophyletic clade that is only distantly related to a *pmmo* (particulate methane monooxygenase); however, this clade contains no currently validated reference sequences, and we refer to its function as “unknown”. Proteomics confirmed the expression of *phmo*, specifically subunits a and b (Fig. 3, Fig. 4) in the presence of *n-*pentane. The two *phmo* genes for which peptides were detected in OOV belong to a sequence from OOV-MAG in the “unknown” clade of *phmos* and one clade containing reference sequences with a demonstrated affinity for ethane and butane. The only detected hydrocarbon monooxygenase in SV-MAG is the *phmo,* supporting the hypothesis that this enzyme functions on *n-*pentane. *AlkB*, a gene known to function on medium to long-chain alkanes, was found encoded in the MAG-OOV; however, no peptides were observed in the proteomics analyses (Fig. 3). Still, given the minimal sample size analyzed for proteomics and the potential for false negatives due to e.g. ionization and extraction efficiencies, we do not exclude the possibility that *alkB* could also be active in these samples and used to consume *n-*pentane by the OOV.

The second step in the consumption of *n-*pentane is the conversion of pentanol to an aldehyde. In many bacteria that oxidize alcohols, this reaction is catalyzed by pyrroloquinoline quinone-dependent alcohol dehydrogenases (*pqq-adh*). We found genes encoding *pqq-adh* in both *Cycloclasticus* MAG variants and proteomic expression of PQQ-ADH in OOV-MAG samples. (Fig. 3, Supplementary Information Fig. S2). None of the *pqq-adh* genes formed a monophyletic clade with reference sequences known to act on methanol, providing evidence against methane metabolism in SV and OOV. In the third step of *n-*pentane consumption, the aldehydes are oxidized to carboxylic acids, which could be achieved via a tungsten-containing aldehyde ferredoxin oxidoreductase (*aor*), known to use short-chain alkane-derived aldehydes as their substrate [15, 71]. This conversion can also be performed by *pqq-adh*, as activity on aldehydes has been confirmed with reference sequences related to those encoded by SV and OOV (Supplementary Information Fig. S2). Here, pentanoate is likely beta-oxidized using acyl-CoA dehydrogenase and enoyl-CoA hydratase and shunted into central carbon metabolism via the citric acid cycle (Fig. 3).

### Differences in variant metabolic potential

**Figure 5:**
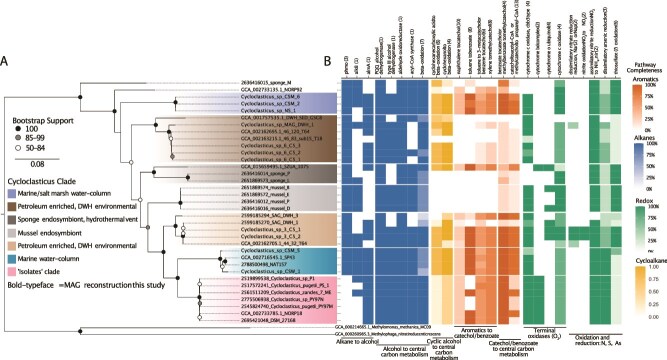
Phylogeny and capabilities for hydrocarbon consumption of *Cycloclasticus*. **A** phylogeny constructed using 16 ribosomal proteins, subclades are designated based on relative distance from the root and supported by average nucleotide identity (supplementary information, Fig. S5). Branch lengths represent the number of substitutions per site. **B** completeness of select metabolic pathways relating to alkane, aromatic hydrocarbon, cycloalkane metabolism, and redox. The number of genes considered in calculating pathway completeness is shown in parentheses.

The metabolic capabilities of the SV-MAG and OOV-MAG differ substantially (Fig. 3, Fig. 4). The OOV-MAG encodes for general hydrocarbon metabolism that includes the nearly complete pathway for toluene consumption via the toluene monooxygenase conversion of toluene to benzoate (seven of eight genes), benzoate conversion to catechol (three of four genes), and the catechol metacleavage to acetyl-CoA which enters the tricarboxylic acid cycle (13 of 13 genes). The OOV-MAG also encodes toluene 2-monooxygenase, which converts benzene to catechol (six of six genes) that can also be shunted through the same catechol meta-cleavage pathway as toluene to form acetyl-CoA (13 of 13 genes) and enter the tricarboxylic acid cycle. The OOV could also use the toluene-2 monooxygenase system to convert toluene to 3-methylcatechol (six of six genes) and then convert 3-methylcatechol to acetyl-CoA and shunt to the tricarboxylic acid cycle (three of five genes). Furthermore, the OOV-MAG encodes for *alkB* (one of one gene), which is commonly used by other organisms for consumption of long-chain alkanes via beta-oxidation (OOV encodes seven of seven genes), resulting in propionyl-CoA and acetyl-CoA, which are also incorporated into the tricarboxylic acid cycle. Neither OOV-MAG or SV-MAG (or any other *Cycloclasticus* MAGs analyzed in this study) encode a complete canonical naphthalene degradation pathway (naphthalene 1,2, dioxygenase is missing from all genomes/MAGs), yet the strain *Cycloclasticus* SP-1 has been experimentally validated to use naphthalene as a sole carbon source (Fig. 5B) [72]. *Cycloclasticus* SP-1 and OOV-MAG encode three of ten genes for naphthalene degradation, which indicates that OOV-MAG can also likely metabolize naphthalene, whereas SV encodes zero of ten genes.

Overall, the SV-MAG lacks many metabolic pathways for longer-chain alkanes and aromatic compounds compared to the OOV-MAG, seemingly limiting its hydrocarbon metabolism potential (Fig. 4, Fig. 5B). These observed differences are consistent with SV specialization on short-chain, aqueous-soluble alkanes and biogeography that includes seeding from the petroleum-rich source region in the Northern GOM. The genomic capacity for catabolism of multiple hydrocarbon classes in the OOV-MAG is consistent with an ability to capitalize on diffuse hydrocarbon sources that expand beyond natural seeps, including atmospheric deposition, terrestrial runoff, biogenic inputs, and oil spills. This enhanced capacity in OOV is consistent with an expanded biogeographic range relative to SV, which appears to be more highly reliant on substrate sourced from natural seepage (Supplementary Dataset S5 and Supplementary Information Fig. S9).

### Anaerobic metabolism in *Cycloclasticus*

Anaerobic metabolism has yet to be observed in *Cycloclasticus,* and it remains unknown how these bacteria could contribute to hydrocarbon cycling in oxygen minimum zones or anoxic sediments. Here, we show the OOV-MAG of *Cycloclasticus* exhibits adaptations for life without oxygen, including the occurrence of genes for respiratory nitrate reductase (*Nar*), as well as a potential linkage to thiosulfate metabolism (Fig. 4). In OOV-MAG, we identify a complete canonical *nar* operon (*narGHJI*) encoding: (i) the α subunit responsible for catalyzing NO_3_^−^ reduction to NO_2_^−^ (*narG*); (ii) the iron and sulfur-containing β subunit (*narH*) that transfers electrons to the molybdenum cofactor of *narG*; (iii) the *narJ* chaperone used in enzyme formation, and (iv) the transmembrane γ subunit (*narI*) involved in electron transfer from membrane quinols to *narH*. Phylogenetic placement of *Cycloclasticus narG s*equences also confirms the relation to *narG* reference sequences (Supplementary Information Fig. S1).

OOV-MAG also contains the *sox* operon (*soxCDXYZAB*), which encodes periplasmic sulfur-oxidizing proteins (Fig. 4). This operon can be used as a means of detoxification in some *Gammaproteobacteria* [8]; however, we do not exclude the possibility that *Cycloclasticus* could employ a lithoheterotrophic strategy. The use of thiosulfate to supplement heterotrophy is a strategy that has been demonstrated in other *Proteobacteria* and could be useful in seeps and other benthic environments [73]. It is unclear how members of *Cycloclasticus* may access *n-*pentane in the absence of oxygen. No enzymes related to alkyl succinate synthase were detected. Multiple putative hits for the DMSO protein superfamily were detected, and this superfamily encompasses a variety of functions, including the anaerobic alkane degradation enzyme, alkane C2 methylene hydroxylase; however, OOV-MAG sequences do not form a monophyletic clade with reference sequences of this function (data not shown).

### Deepwater Horizon *Cycloclasticus*

The microbial response to the 2010 Deepwater Horizon (DWH) blowout in the GOM induced blooms of *Cycloclasticus* in the deep ocean, driven by large-scale intrusions of dissolved hydrocarbons [74]. These DWH blooms included multiple *Cycloclasticus* 16S rRNA gene sequence variants, prompting us to investigate whether the seep variant (SV) and open-ocean variant (OOV) were among them. We analyzed the 16S rRNA gene content and performed high-throughput sequencing on a sample collected while active flow occurred from the wellhead into the GOM. At the sampling depth, an oxygen anomaly characteristic of the respiratory response associated with the DWH subsurface intrusions was detected [75] (Supplementary Information Fig. S6). Initial analysis of the microbial community via the V4 region (252 bp) of the 16S rRNA gene revealed that the SV-MAG was identical to the dominant *Cycloclasticus* variant in the DWH sample and OOV-MAG matched the second most abundant *Cycloclasticus* single nucleotide variant (Fig. 2A).

Using read-recruitment of metagenomic sequences from the same sample, we find that the fraction of the SV-MAG covered by the DWH reads spans 98% of the MAG with ~180X coverage. The OOV-MAG is 100% covered from reads mapped from the DWH sample with ~21X coverage (Supplementary Dataset S5). We reconstructed a high-quality metagenome, here named “MAG_DWH_1”, which is 94% complete and 3.3% redundant. Upon expanding our analysis to the full-length 16S rRNA gene (as opposed to the V4 region in Fig. 2), we find that the SV-MAG is 99.5% identical to MAG_DWH_1. Through a phylogenomic analysis of 16 RPs, we find MAG_DWH_1 forms a monophyletic clade with SV-MAG (Fig. 5A, Supplementary Information Fig. S4). For comparison, we also drew from our previously published SAGs from DWH, which are 71%, 49%, and 46% complete and herein referred to as “SAG_DWH_3”, “SAG_DWH_1”, and “SAG_DWH_2” [15]. We find that “SAG_DWH_1” and “SAG_DWH_3” are closely related to OOV-MAG, whereas “SAG_DWH_2” appears to be related to SV-MAG (Supplementary Information Fig. S4). For the relation of SV and OOV to the SAGs and MAG_DWH_1, we also find supporting evidence in the analysis of Average Nucleotide Identity and the 16S gene rRNA phylogeny (Supplementary Information Fig. S4 and S5). These results indicate a previously unrecognized distinction in the microbial response to the DWH event – that SV-like *Cycloclasticus* may have responded specifically to the highly abundant soluble *n*-alkanes. In contrast, OOV-like *Cycloclasticus* may have responded to soluble *n*-alkanes and other components, including benzene and toluene.

To further assess the ecological relevance of SV and OOV *Cycloclasticus* to DWH, we compared the similarities in the *phmo* phylogenetic placement between SV- and OOV-MAGs and previously published transcripts from DWH subsurface plumes (Fig. 4) [76]. These results indicate that *phmo* genes most closely related to SV and OOV *Cycloclasticus* were expressed at high relative abundance during the DWH event, consistent with data showing the rapid microbial response by *Cycloclasticus* to short-chain n-alkanes (but not methane) concurrent with active discharge [74]. The pulse of bacterial growth in the deep ocean from the DWH event has been estimated at >10^23^ cells, with a substantial fraction being SV *Cycloclasticus* [8]. We, therefore, questioned if this level of ecological disturbance might have structured the hydrocarbon-degrading community in the GOM through 2015 when samples for this work were collected. More data is needed to assess this hypothesis rigorously. Other researchers did find that methanotrophic biomass remained elevated in the years following the DWH event, perpetuating elevated methanotrophic activity above the background levels existing before the disaster [77]. Therefore, it remains possible that the Cycloclasticus observed in our *n-*pentane incubations was poised to bloom five years following the spill due to some form of memory effect from the large influx of biomass caused by the disaster.

### Hydrocarbon metabolism across *Cycloclasticus*

To understand how hydrocarbon metabolic capability within Cycloclasticus relates to ecological and evolutionary patterns, we reconstructed *Cycloclasticus* MAGs from various environments using publicly available datasets (Supplementary Dataset S4). This effort resulted in eight high-quality MAGs with completion of >80% and < 2% redundancy. These eight MAGs are in addition to the five pentane MAGs and the one DWH-MAG already discussed and includes one from the uncontaminated North Sea “NS_1”, six from a coastal salt marsh in Skidaway Island, Georgia, “CSM_1”, “CSM_2”, “CSM_3”, “CSM_4”, “CSM_5”, and “CSM_6”, and one MAG from coastal seawater near Pivers Island, North Carolina “CSW_1”. The 14 MAGs reconstructed for this study, along with other publicly available genomes, were used to form a phylogenomic tree of all *Cycloclasticus* (Fig. 5A)*.* Each genome was then scanned for hydrocarbon-related pathways of interest and other metabolic functions related to energy generation (Fig. 5B).

From the phylogenetic analysis of RPs alongside metabolic data, we observe distinct strategies by each major clade within the *Cycloclasticus* genera (Fig. 5B). All cultivated *Cycloclasticus* are very closely related to each other (Fig. 5A, bottom clade, denoted “isolates”). We found no evidence of genes for consuming short-chain alkanes within this clade. This is a major bias in our understanding of *Cycloclasticus*, because all other *Cycloclasticus* MAGs analyzed contained *phmo* genes. We also observe two water-column clades from uncontaminated seawater that harbor diverse pathways for short-chain and long-chain alkanes, as well as near-complete pathways for naphthalene and xylene, and complete pathways for toluene and benzene consumption. Altogether, we find a minimum of seven clades within the *Cycloclasticus*, seemingly unified as marine organisms that grow from aqueous soluble hydrocarbons. One key factor distinguishing the clades is the evolved preference to access certain classes of aqueous soluble hydrocarbons and not others.

## Conclusion

Our study provides genomic and proteomic evidence for *n-*pentane metabolism by free-living members of the *Cycloclasticus* genus in contrasting oceanic regimes, one with prolific natural seep influence and another farther removed from prolific seepage. By comparing *Cycloclasticus* genomes and MAGs, we show that the hydrocarbon metabolism within this genus is not limited to PAH degradation, with genomic variability enabling different ecotypes to access different ecological niches and structural classes of hydrocarbons. The apparent commonality among *Cycloclasticus* is not the ability to consume aromatic hydrocarbons, as the genus name suggests, but rather a metabolic specialization among the subset of hydrocarbons that exhibit aqueous solubility in marine settings.

Our findings build on previous research that identified contrasting strategies among *Cycloclasticus* members: cultivated strains that rely exclusively on aromatic hydrocarbons and mussel and sponge symbionts that primarily consume short-chain alkanes. Expanding on these observations, we identify distinct clades of free-living *Cycloclasticus* characterized by substrate specializations and varied geographic distributions. The seep variant (SV) clade selectively targets short-chain alkanes via the *phmo* pathway, whereas the open-ocean variant (OOV) clade can also target short-chain alkanes but demonstrates broader hydrocarbon versatility, including the ability to degrade aromatics and longer-chain alkanes, as well as the capacity for anaerobic metabolism. This metabolic versatility likely provides *Cycloclasticus* with an adaptive advantage in transient natural seep environments and access to hydrocarbon sources beyond seeps.

The specialization of SV *Cycloclasticus* on short-chain alkanes is intriguing because of the apparent ecological risk. This strategy relies on a consistent supply of short-chain alkanes, which are supplied almost exclusively from petroleum-rich environments such as seeps or spills. Given the geographic limitations on petroleum seepage and the transient nature of discharge, it is surprising that specialist bacterioplankton have evolved into this niche. Nevertheless, the results of our experiments and the evident success of SV *Cycloclasticus* during DWH disaster indicate that such specialization results in a viable ecological strategy. The success of SV *Cycloclasticus* is likely related to rapid cellular respiration that enables competitive growth upon exposure to the substrate.

The insight gained from this work provides a new vantage for considering the deep ocean microbial response to hydrocarbon discharge during DWH. SV *Cycloclasticus* was well adapted to bloom in response to the massive intrusions of aqueous-soluble *n*-alkanes that accompanied this event. The OOV *Cycloclasticus* may have engaged in direct competition by consuming these same compounds, but they may have also accessed other compounds in parallel or in sequence. This work goes beyond the DWH and provides a predictive capacity for understanding the ocean’s response to future industrial incidents on a variety of scales, such as a rupture of a subsea pipeline or the sinking of a tanker vessel carrying gas condensate, light crude oil, or diluted bitumen; or another well blowout.

## Supplementary Material

Supplemental_Information_11_20_ismejo_wrae247

Supplementary_Dataset_S1_ismejo_wrae247

Supplementary_Dataset_S2_ismejo_wrae247

Supplementary_Dataset_S3_ismejo_wrae247

Supplementary_Dataset_S4_ismejo_wrae247

Supplementary_Dataset_S5_ismejo_wrae247

Supplementary_Dataset_S6_ismejo_wrae247

Supplementary_Dataset_S7_ismejo_wrae247

## Data Availability

The datasets generated during and/or analyzed during the current study are available in the NCBI repository under the accessions PRJNA698236, SRR29495059- SRR29495064, ASM4074301v1, ASM4074303v1, ASM4074306v1, ASM4074308v1, ASM4074313v1, and ASM4074315v1. The mass spectrometry data are deposited to the Proteome Xchange Consortium via the PRIDE partner repository with the dataset identifier PXD022428.
