# Supplementary material for: Hydrocarbon metabolism and petroleum seepage as ecological and evolutionary drivers for Cycloclasticus
Source: ISME J. 2024 Dec 18;19(1):wrae247. doi: 10.1093/ismejo/wrae247 (PMC12510462; doi:10.1093/ismejo/wrae247)
Supplement: Supplemental_Information_11_20_ismejo_wrae247 [file supplemental_information_11_20_ismejo_wrae247.pdf]

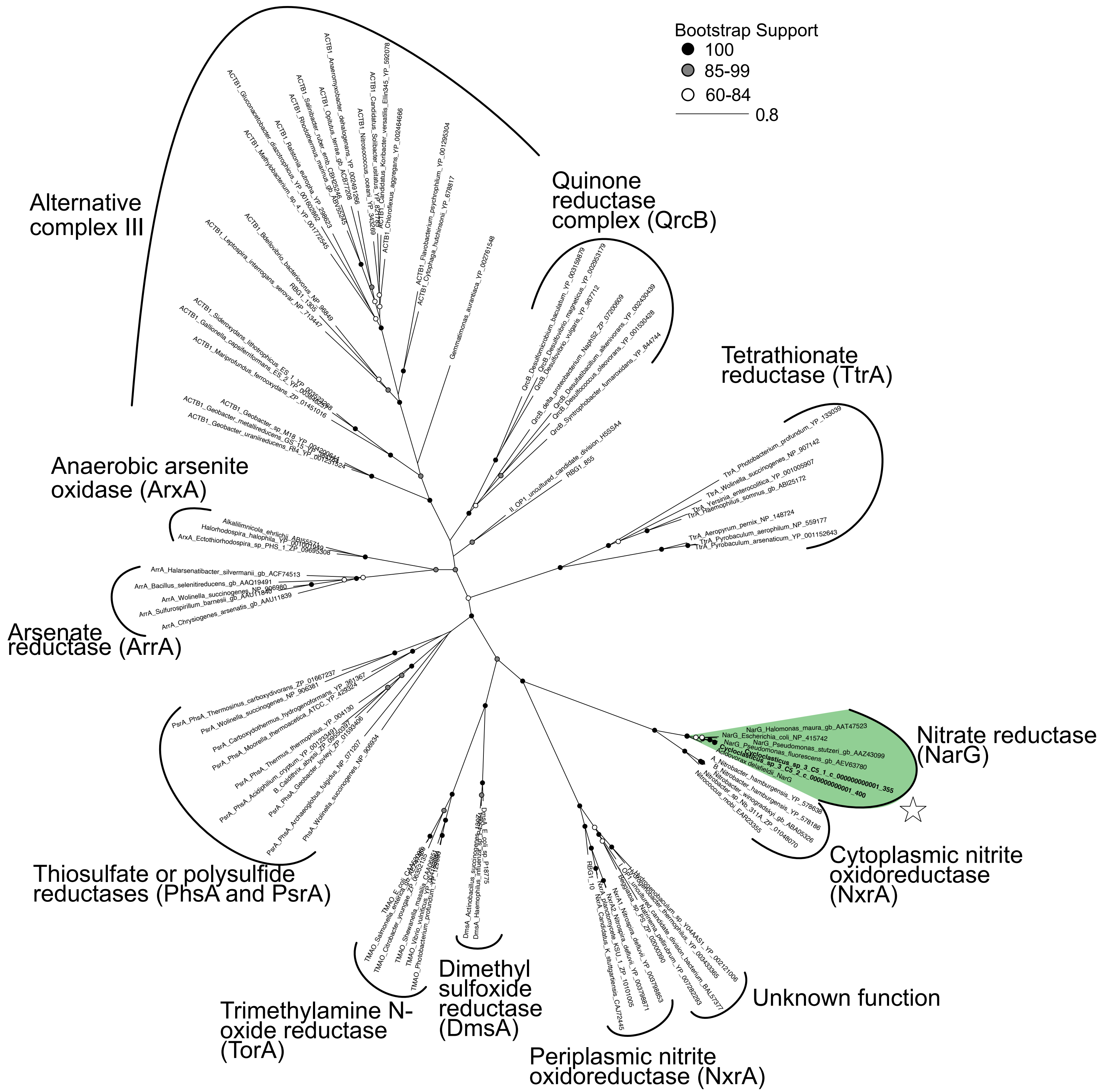

Supplemental Data Figure S1. Maximum-likelihood phylogenetic tree with scale bar of substitutions per site of DMSO reductase superfamily modeled after [1]. NarG sequences from Cycloclasticus OOV variant in bold and noted with star symbol. See Supplementary Dataset S6 for accessions and Supplementary Dataset S7 for sequences.

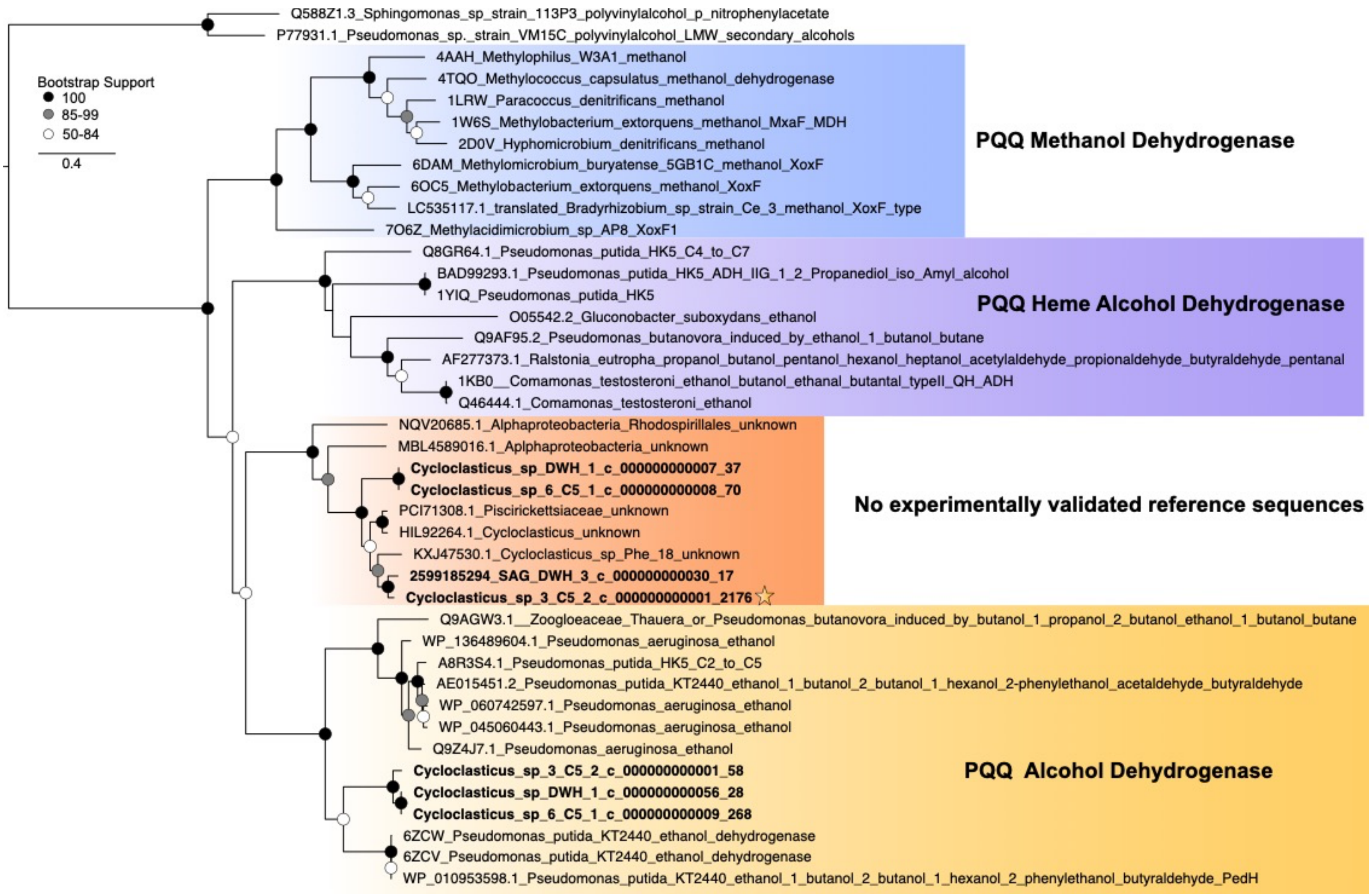

Supplemental Figure S2. Maximum-likelihood phylogenetic tree of PQQ-dependent alcohol dehydrogenase sequences rooted to polyvinylalcohol reference sequences. Experimentally validated reference sequences are annotated with known substrate activity. Sequence denoted with a star was observed in proteomic samples from pentane enrichment. No experimentally validated reference sequences present in the orange clade. See Supplementary Dataset S6 for accessions and Supplementary Dataset S7 for sequences.

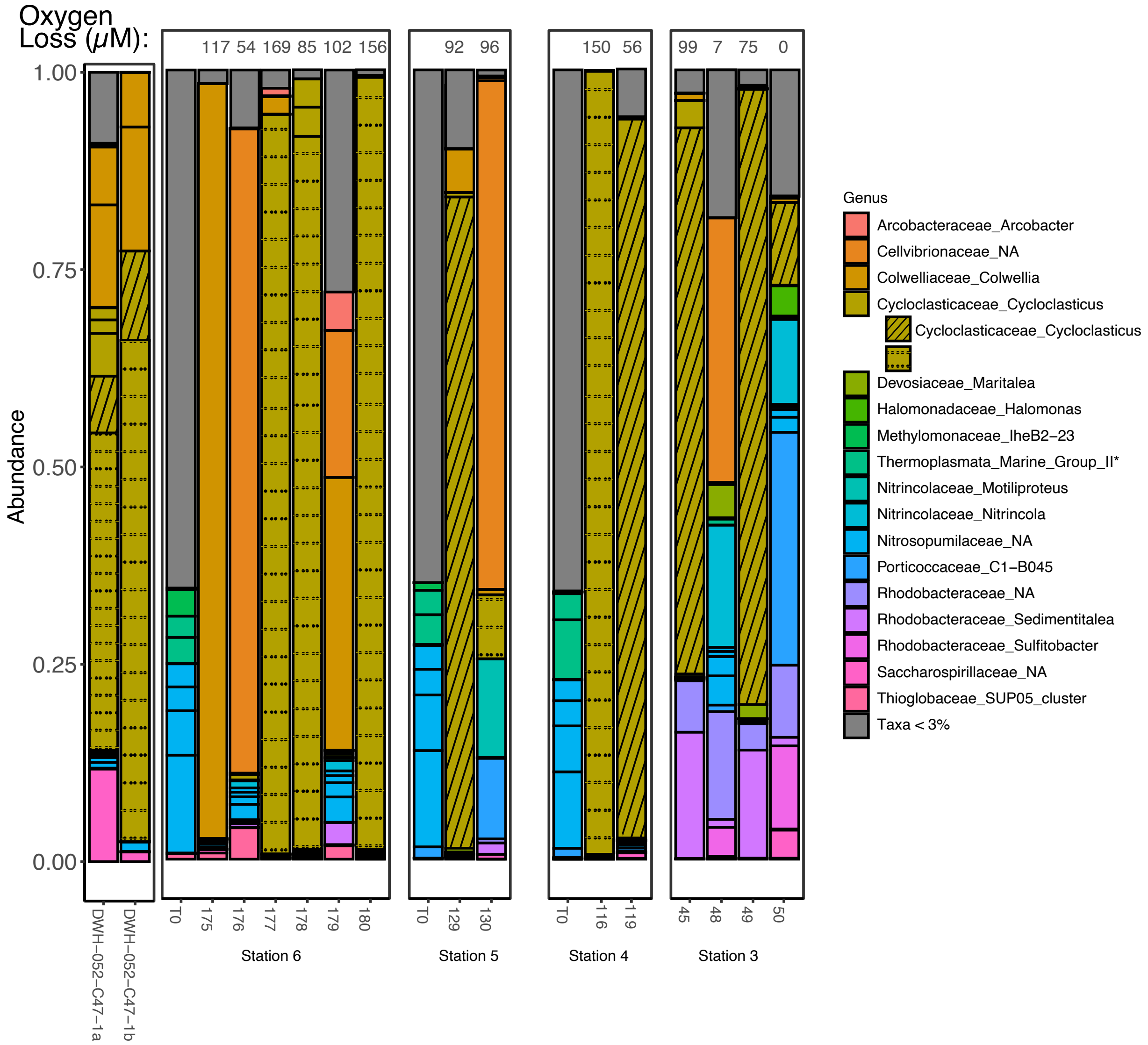

Supplemental Figure S3. 16S rRNA community analysis of V4 region. Sample 119, 48, and 50 are “non-bloom” samples.

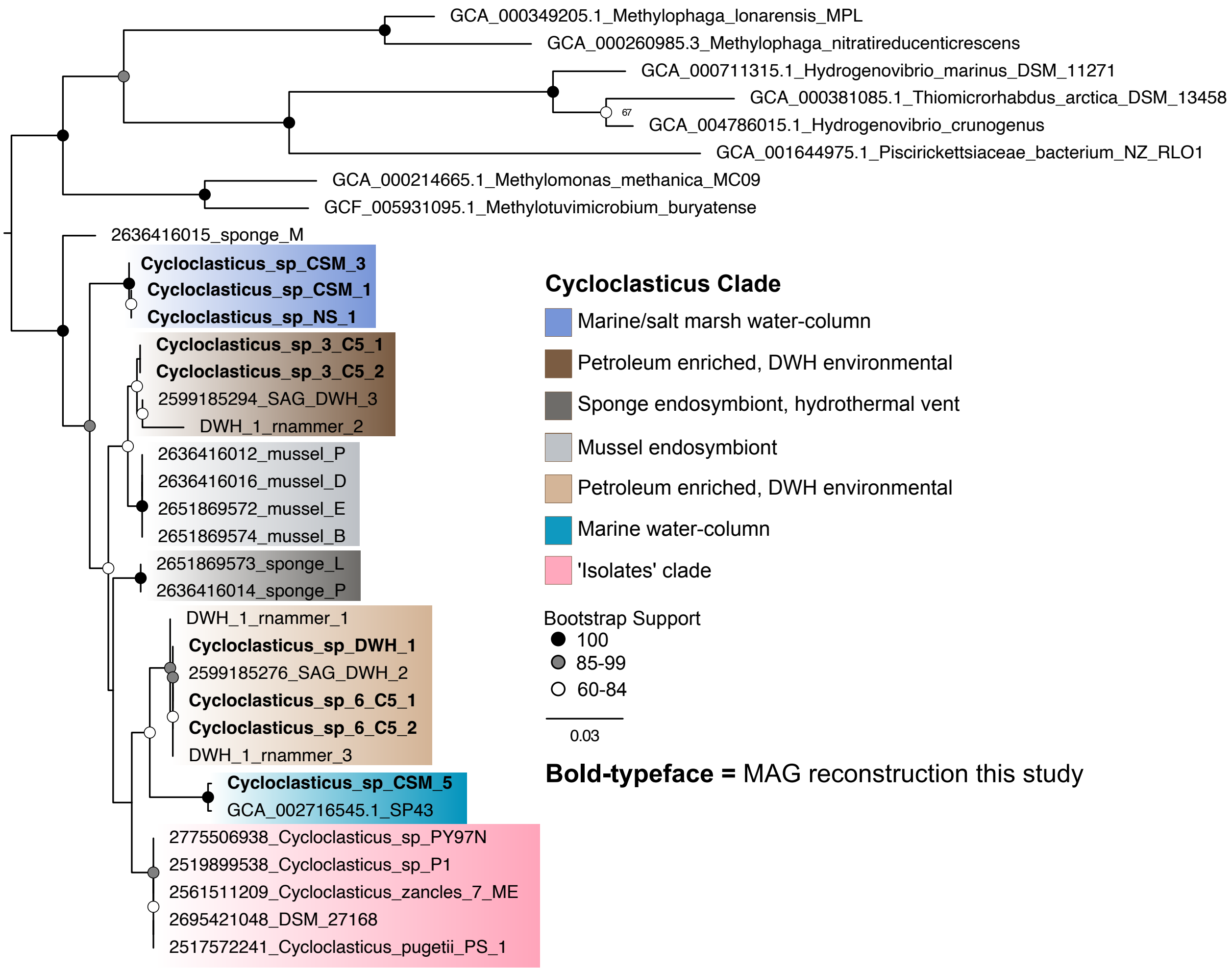

Supplemental Figure S4. Maximum-likelihood phylogenetic tree of 16S rRNA rooted to representatives from *Piscirickettsiaceae* and *Methylococcaceae* family. "_rnammer" denotes 16S sequences recovered from assembled contigs prior to binning for the DWH sample. Alignment includes full length 16S rRNA gene 1,547 amino acid residues. See Supplementary Dataset S6 for accessions and Supplementary Dataset S7 for sequences.

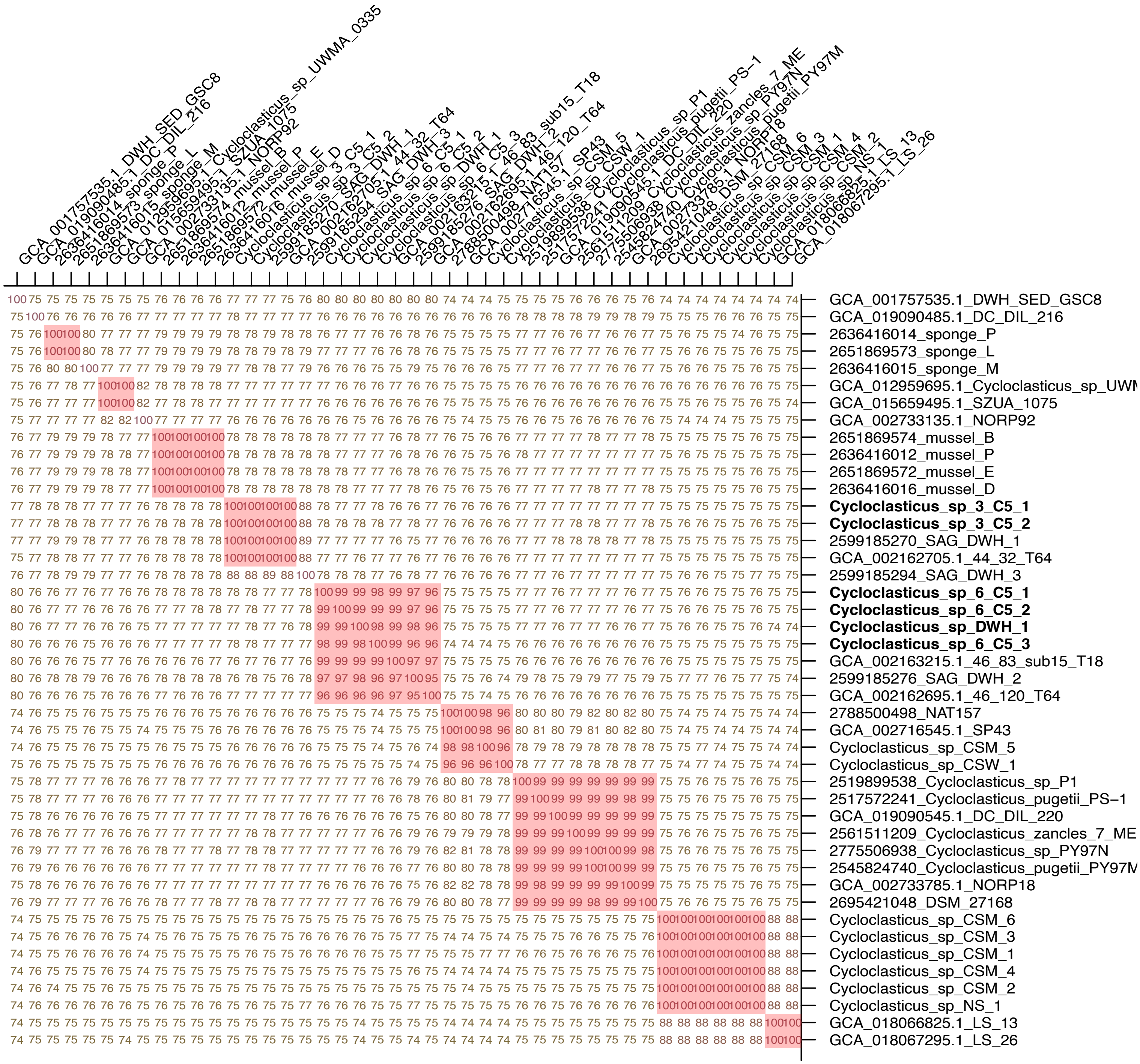

Supplemental Figure S5. Average nucleotide identity of *Cycloclasticus* genomes. SV and OOV are highlighted in bold.

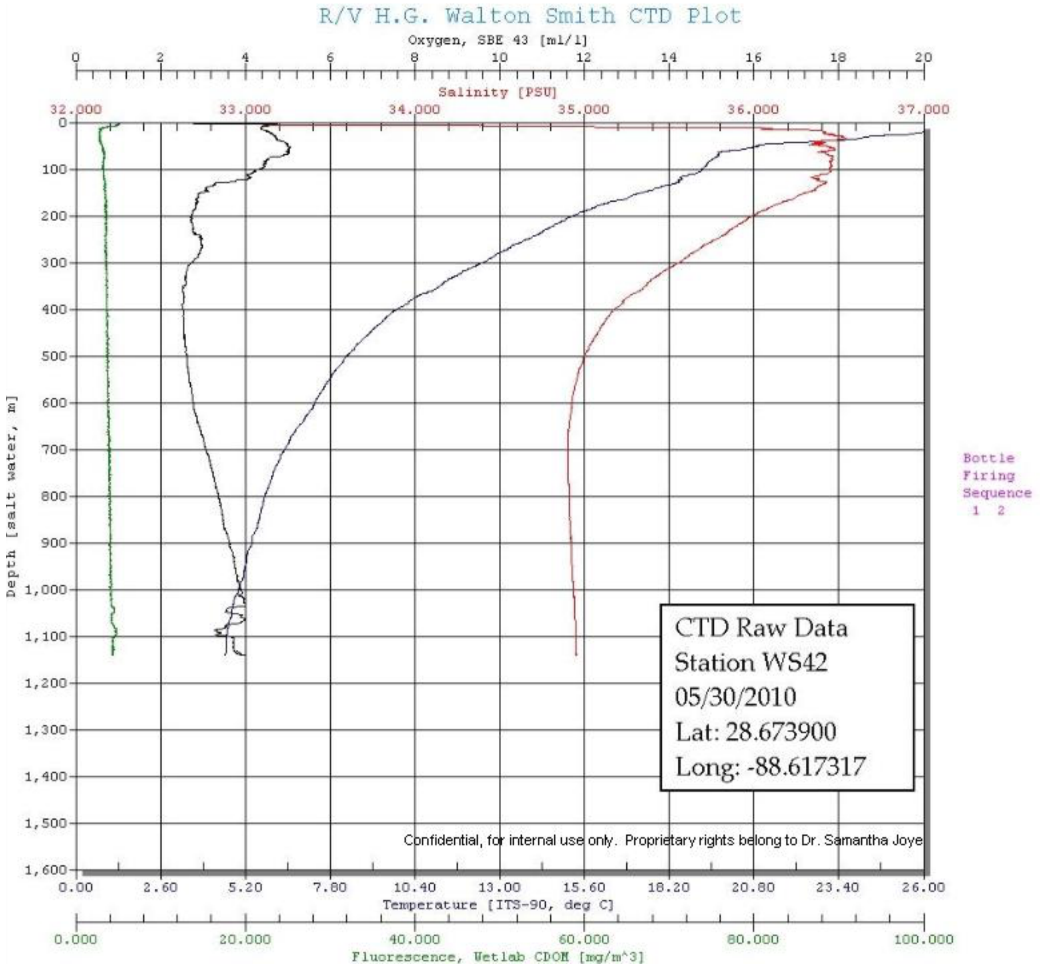

Supplemental Figure S6. CTD profile from cast during the DWH event. DWH sample analyzed in this study was collected at 1,090m by Dr. Molly Redmond. [2]

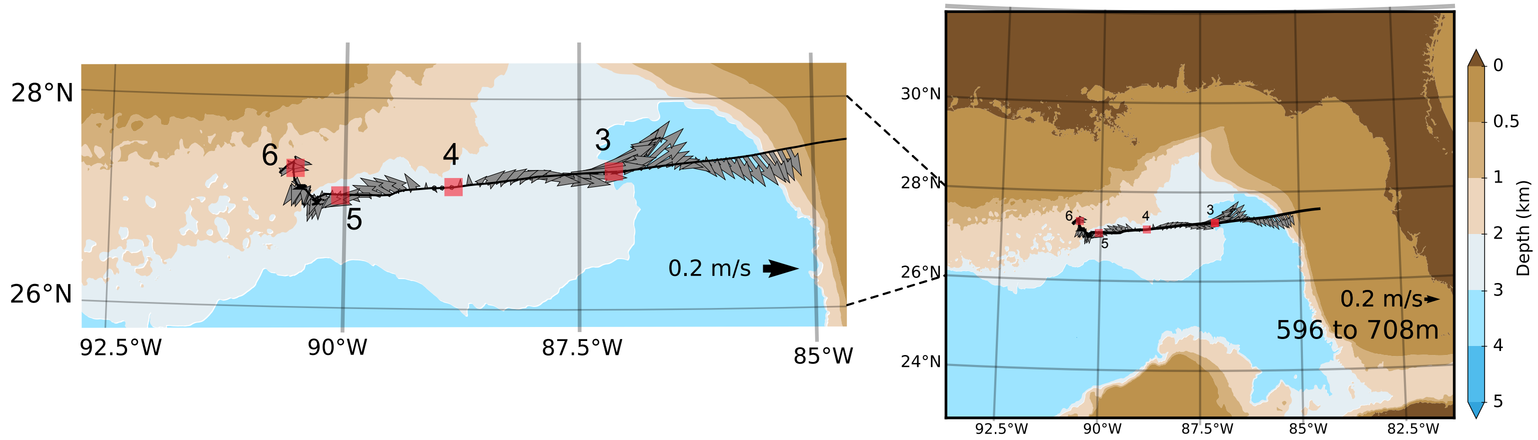

Supplemental Data Figure S7. ADCP data showing deep ocean currents over the course of the GOM sampling expedition (RV Atlantis, 2015).

a

phmo 95% AAI Clusters among *Cycloclasticus* sp.

**Cluster 1: Cycloclasticus_sp_6_C5_1_c_000000000007_32**	**Amino Acid Identity (%)**
Cycloclasticus_sp_6_C5_2_c_000000000004_18	100.00
Cycloclasticus_sp_6_C5_3_c_000000000010_33	99.19
Cycloclasticus_sp_DWH_1_c_000000000072_23	100.00
GCA_002162695.1_46_120_T64_c_000000000010_18	99.19
GCA_002163215.1_46_83_sub15_T18_c_000000000090_33	98.79
**Cluster 2: Cycloclasticus_sp_6_C5_1_c_000000000008_249**	**Amino Acid Identity (%)**
Cycloclasticus_sp_6_C5_2_c_000000000029_31	99.26
Cycloclasticus_sp_6_C5_3_c_000000000003_140	99.26
Cycloclasticus_sp_DWH_1_c_000000000077_3	100.00
GCA_002162695.1_46_120_T64_c_000000000014_12	99.26
**Cluster 3: 2636416012_mussel_P_c_000000000101_6**	**Amino Acid Identity (%)**
2636416016_mussel_D_c_000000000078_14	99.64
2651869572_mussel_E_c_000000000178_6	100.00
2651869574_mussel_B_c_000000000024_6	100.00
**Cluster 4: 2599185270_SAG_DWH_1_c_000000000007_34**	**Amino Acid Identity (%)**
Cycloclasticus_sp_3_C5_1_c_000000000001_434	100.00
Cycloclasticus_sp_3_C5_2_c_000000000001_479	100.00
GCA_002162705.1_44_32_T64_c_000000000071_28	100.00
**Cluster 5: 2788500498_NAT157_c_000000000008_267**	**Amino Acid Identity (%)**
Cycloclasticus_sp_CSM_5_c_000000000018_6	100.00
Cycloclasticus_sp_CSW_1_c_000000000006_41	99.27
GCA_002716545.1_SP43_c_000000000001_6	100.00
**Cluster 6: 2599185294_SAG_DWH_3_c_000000000035_18**	**Amino Acid Identity (%)**
Cycloclasticus_sp_3_C5_1_c_000000000001_1123	99.25
Cycloclasticus_sp_3_C5_2_c_000000000001_1167	99.25
GCA_002162705.1_44_32_T64_c_000000000076_5	99.25
**Cluster 7: Cycloclasticus_sp_CSM_1_c_000000000010_39**	**Amino Acid Identity (%)**
Cycloclasticus_sp_CSM_2_c_000000000015_14	100.00
Cycloclasticus_sp_CSM_6_c_000000000063_39	100.00
Cycloclasticus_sp_NS_1_c_000000000002_39	100.00
**Cluster 8: 2636416012_mussel_P_c_000000000113_37**	**Amino Acid Identity (%)**
2636416016_mussel_D_c_000000000026_39	100.00
2651869572_mussel_E_c_000000000058_90	100.00
2651869574_mussel_B_c_000000000038_37	100.00
**Cluster 9: Cycloclasticus_sp_3_C5_1_c_000000000001_285**	**Amino Acid Identity (%)**
Cycloclasticus_sp_3_C5_2_c_000000000001_330	100.00
GCA_002162705.1_44_32_T64_c_000000000041_4	100.00
**Cluster 10: 2636416014_sponge_P_c_000000000077_102**	**Amino Acid Identity (%)**
2651869573_sponge_L_c_000000000067_173	100.00

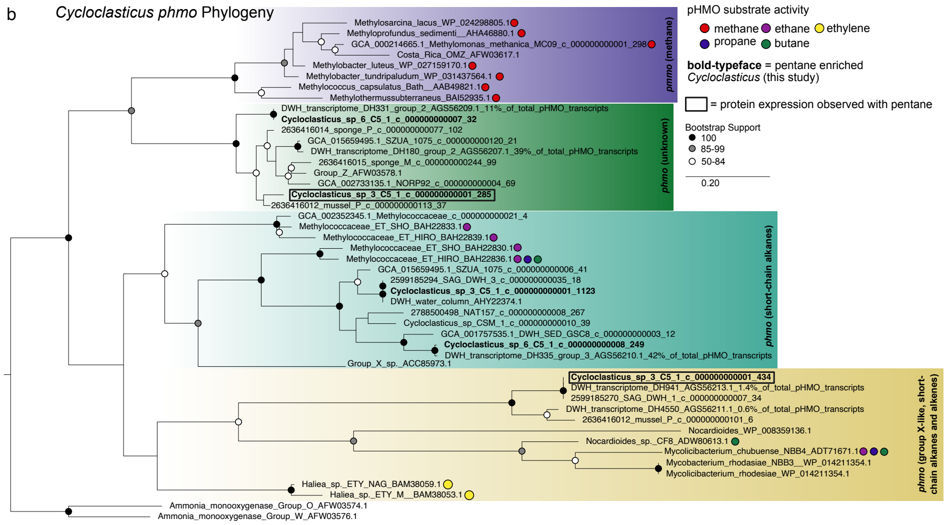

Supplemental Data Figure S8. Phylogeny of *phmo* subunit A protein sequences. **a** Description of *phmo* clusters containing 95% amino acid identity. Amino acid identity to the representative sequence is noted. The representative sequence for each cluster is colored according to the phylogenetic placement. **b** Maximum likelihood tree of representative sequences is drawn to scale, with branch lengths representing the number of substitutions per site. Bootstrap values below 50% are not shown. Each major clade is color-coded for readability, with purple representing *pmmo* with activity on methane, the green clade is "unknown" and lacks any known substrate specificity, the cyan clade representing group X *phmo* (ethane/ethylene, propane, and butane activity), and the yellow clade are group-X like (ethane, propane, and butane activity). Sequences from the pentane-enriched *Cycloclasticus* MAGs are in bold, boxed values indicate *phmo* detected in proteomic data. See Supplementary Dataset S6 for accessions and Supplementary Dataset S7 for sequences.

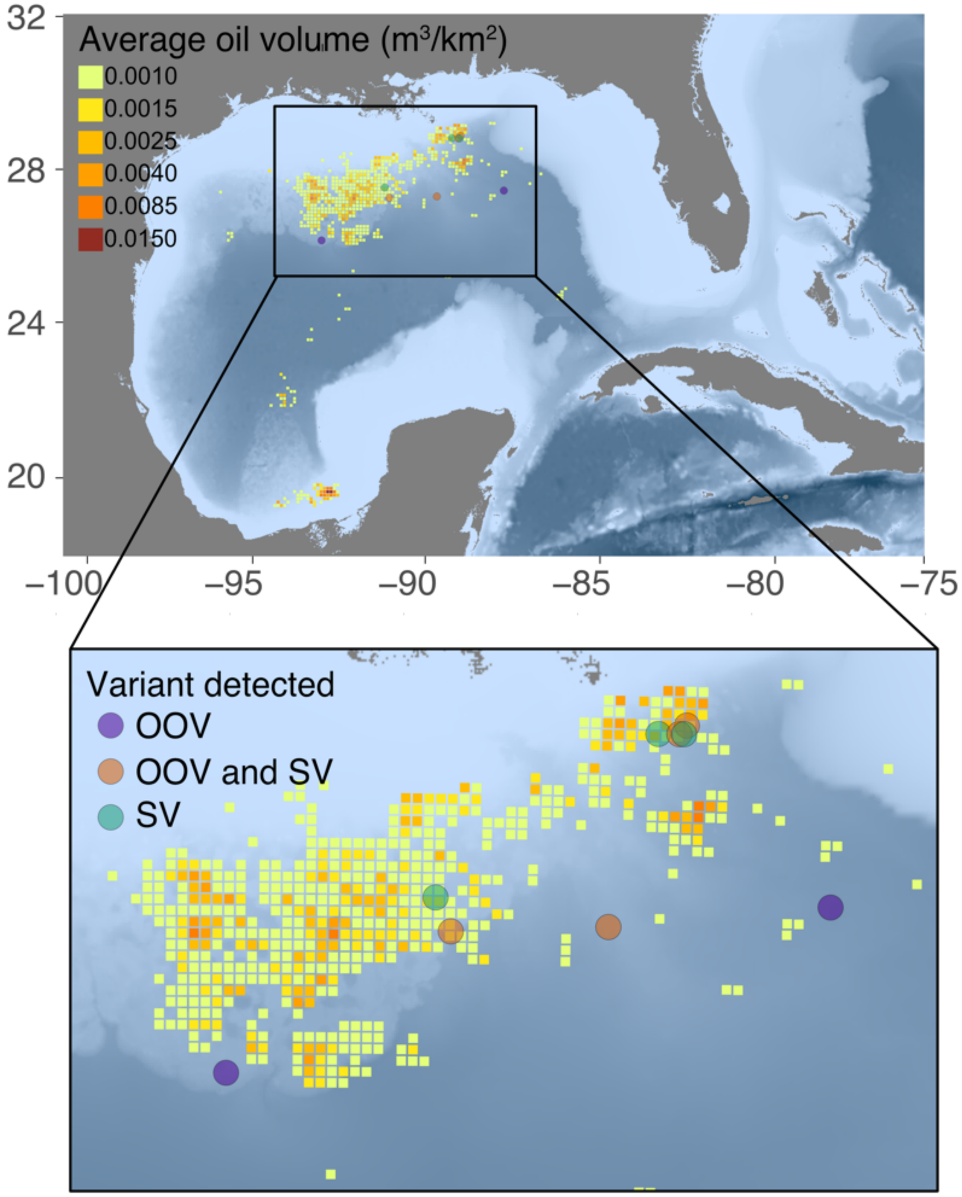

Supplemental Data Figure S9. Read-recruitment to *Cycloclasticus* OOV-MAG and SV-MAG across public data and this study. Genome is present if reads recruit to over 20% of the genome, and the expected breadth and breadth are within 20% of each other, indicating recruited reads are randomly distributed throughout the genome instead of a small number of genes.

References

1. Castelle CJ, Hug LA, Wrighton KC, Thomas BC, Williams KH, Wu D, et al. Extraordinary phylogenetic diversity and metabolic versatility in aquifer sediment. *Nat Commun* 2013; **4**.
2. National Oceanic and Atmospheric Administration *R/V Walton Smith CTD profile data*. Available at https://www.ncei.noaa.gov/data/oceans/DeepwaterHorizon/Ship/Walton_Smith/ORR/Cruise_01/CTD/Products/. Accessed 10 Jun 2024.
